# Supplementary material for: Identifying the Pressure Points of Acute Cadmium Stress Prior to Acclimation in Arabidopsis thaliana
Source: Int J Mol Sci. 2020 Aug 28;21(17):6232. doi: 10.3390/ijms21176232 (PMC7503646; doi:10.3390/ijms21176232)
Supplement: Supplementary file 1 [file ijms-21-06232-s001.pdf]

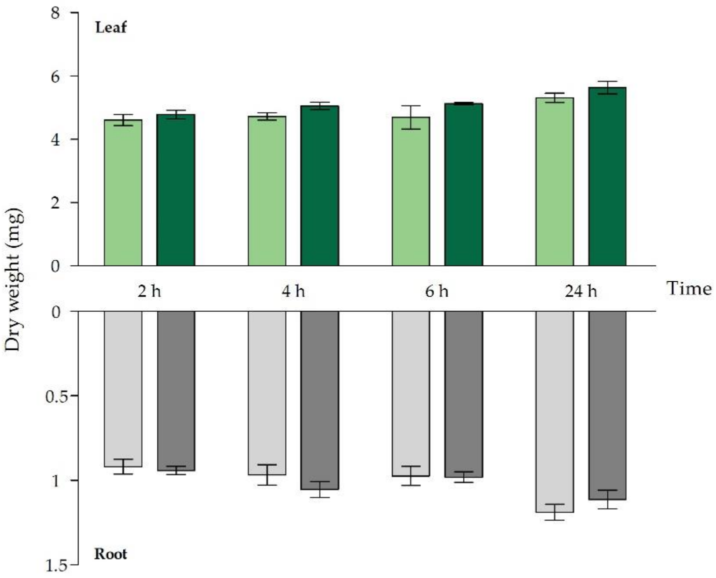

Figure S1 Rosette (green bars) and root (gray bars) dry weight (mg) of *Arabidopsis thaliana* plants grown under control conditions (0 μM $CdSO_4$, light bars) or exposed to 5 μM $CdSO_4$ (dark bars) during 2 h, 4 h, 6 h and 24 h after 3 weeks of growth. For each time point, data represent the mean ± S.E. of 5 biological independent replicates. No significant differences (t-test: $p < 0.05$) were observed between control and exposed plants, within each time point.

Table S1 Percentage reduced glutathione (GSH) in leaves and roots of *Arabidopsis thaliana* plants grown under control condition (0 μM $CdSO_4$) or exposed to 5 μM $CdSO_4$ during 2 h, 4 h, 6 h and 24 h after 3 weeks of growth. For each time point, data represent the mean ± S.E. of 4 biological replicates. Significant differences (t-test: $p < 0.05$) between control and exposed plants, within each time point, are indicated with an asterisk (*).

		Reduced GSH (%)			
	**[$CdSO_4$]**	**2 h**	**4 h**	**6 h**	**24 h**
Leaf					
	0 μM	97.80 ± 0.20	97.62 ± 0.43	91.70 ± 0.00	97.20 ± 0.15
	5 μM	97.97 ± 0.25	95.56 ± 0.28	91.71 ± 0.00	99.07 ± 0.10*
Root					
	0 μM	97.15 ± 0.27	97.13 ± 0.81	94.15 ± 1.02	97.97 ± 0.54
	5 μM	98.32 ± 0.33*	99.71 ± 0.41	98.24 ± 0.48*	98.97 ± 0.19

Table S2 Sequence of forward and reverse primers used for gene expression analyses (RT-qPCR) of genes of interest. UTR: untranslated region; x2: primer concentration of 600 nM instead of 300 nM, x3: primer concentration of 900 nM instead of 300 nM.

Genes of interest					
**Gene**	**Locus**	**Forward Primer (5′- 3′)**	**Reverse Primer (5′-3′)**	**Primer Location**	**Amplicon Size**
GSH1	AT4G23100	CCCTGGTGAACTGCCTTCA	CATCAGCACCTCTCATCTCCA	Exon 10 and 11	101 bp
GSH2	AT5G27380	GGACTCGTCGTTGGTGACAA	TCTGGGAATGCAGTTGGTAGC	Exon 1 and 2	101 bp
GR1	AT3G24170	CTCAAGTGTGGAGCAACCAAAG	ATGCGTCTGGTCACACTGC	Exon 15 and 16	101 bp
GGT1	AT4G39640	TAGATGTTCCACCACCAGCA	GGCAATAATGTTGGCTCCTC	Exon 4 and 5	140 bp
ZAT6	AT5G04340	TGACCTGCCTTCTTCTTCGT	GTCCACCAAGAGCCTGGTAA	No introns	113 bp
RBOHC	AT5G51060	TCACCAGAGACTGGCACAATAAA	GATGCTCGACCTGAATGCTC	Exon 6 and 7	101 bp
RBOHD	AT5G47910	AACTCTCCGCTGATTCCAACG	TGGTCAGCGAAGTCTTTAGATTCCT	Exon 1 and 2	91 bp
RBOHF	AT1G64060	GGTGTCATGAACGAAGTTGCA	AATGAGAGCAGAACGAGCATCA	Exon 11 and 12	105 bp
AT1G05340	AT1G05340	TCGGTAGCTCAGGGTAAAGTGG	CCAGGGCACAACAGCAACA	Exon 2 and 3 op compl	91 bp
AT1G19020	AT1G19020	GAAAATGGGACAAGGGTTAGACAAA	CCCAACGAAAACCAATAGCAGA	No introns	92 bp
AT1G57630	AT1G57630	ACTCAAACAGGCGATCAAAGGA	CACCAATTCGTCAAGACAACACC	No introns	91 bp
AT2G21640	AT2G21640	GACTTGTTTCAAAAACACCATGGAC	CACTTCCTTAGCCTCAATTTGCTTC	Exon 1 and 2	91 bp
AT2G43510	AT2G43510	ATGGCAAAGGCTATCGTTTCC	CGTTACCTTGCGCTTCTATCTCC	Exon 1 and 2	91 bp
ZAT12	AT5G59820	GTGCGAGTCACAAGAAGCCTAACA	GCGACGACGTTTTCACCTTCTTCA	No introns	72 bp
RRTF1	AT4G34410	CGGAGCAAGAGCTTTCAGTT	GCGCTTATCACTGTGCTGTC	No introns	109 bp
ACS2	AT1G01480	CATGTTCTGCCTTGCGGATC	ACCTGTCCGCCACCTCAAGT	Exon 3 and 4	91 bp
ACS6 x3	AT4G11280	TTAGCTAATCCCGGCGATGG	ACAAGATTCACTCCGGTTCTCCA	Exon 3 and 4	92 bp
ACO2	AT1G62380	TCTACGTTCGTCACCTCCCTCA	CTCTTACCAAAGTCTTTCATGGCC	Exon 2 and 3	91 bp
ACO4	AT1G05010	CTCCGATGTCCCTGATCTCG	ATCCAGTAGCTCCTCCGACAACT	Exon 2 and 3 compl	91 bp
ERF1 x2	AT3G23240	TCCTCGGCGATTCTCAATTTT	CAACCGGAGAACAACCATCCT	No introns	91 bp
OXI1	AT3G25250	TAGAGGATCGAACCGGAAAG	GACCCTTGATTTCCTCAACG	Exon 2	149 bp
MPK3	AT3G45640	GACGTTTGACCCCAACAGAA	TGGCTTTTGACAGATTGGCTC	Exon 5 and 6	103 bp
MPK6	AT2G43790	TAAGTTCCCGACAGTGCATCC	GATGGGCCAATGCGTCTAA	Exon 5 and 6	101 bp
WRKY33	AT2G38470	TCATCGATTGTCAGCAGAGACG	CCATTCCCACCATTTGTTTCAT	Exon 3 and 4	92 bp

Table S3 Sequence of forward and reverse primers used for gene expression analyses (RT-qPCR) of selected reference genes. Exon jn: exon junction UTR: untranslated region

Reference genes					
**Gene**	**Locus**	**Forward Primer (5′- 3′)**	**Reverse Primer (5′-3′)**	**Primer Location**	**Amplicon Size**
Root					
UBC21	AT5G25760	CTGCGACTCAGGGAATCTTCTAA	TTGTGCCATTGAATTGAACCC	Exon jn 3-4 and exon 4	61 bp
PPR	AT5G55840	AAGACAGTGAAGGTGCAACCTTACT	AGTTTTTGAGTTGTATTTGTCAGAGAAAG	UTR	59 bp
YSL8	AT5G08290	TTACTGTTTCGGTTGTTCTCCATTT	CACTGAATCATGTTCGAAGCAAGT	UTR	61 bp
Leaf					
ACT2	*AT3G18780*	CTTGCACCAAGCAGCATGAA	CCGATCCAGACACTGTACTTCCTT	Exon 2	68 bp
PPR	AT5G55840	AAGACAGTGAAGGTGCAACCTTACT	AGTTTTTGAGTTGTATTTGTCAGAGAAAG	UTR	59 bp
MON1	AT2G28390	AACTCTATGCAGCATTTGATCCACT	TGATTGCATATCTTTATCGCCATC	Exon 13 and 14	61 bp
FBOX	AT5G15710	TTTCGGCTGAGAGGTTCGAGT	GATTCCAAGACGTAAAGCAGATCAA	Exon 1	63 bp
UBQ10	AT4G05320	GGCCTTGTATAATCCCTGATGAATAAG	AAAGAGATAACAGGAACGGAAACATAGT	UTR	61 bp

Table S4 Reverse transcription quantitative PCR (RT-qPCR) parameters according to the Minimum Information for publication of Quantitative real-time PCR Experiments (MIQE) guidelines based on Bustin *et al.* (2009).

	**Sample - Template**
SOURCE	three-weeks-old *Arabidopsis thaliana* roots and leaves grown in hydroponics
METHOD OF PRESERVATION	snap frozen in N_2, long-term storage at -80°
STORAGE TIME	< 6 months
HANDLING	frozen
EXTRACTION METHOD	silica-columns: RNAqueous™ Kit (Ambion, Thermo Fisher Scientific, Waltham, MA, USA)
RNA; DNA-FREE	turbo DNA-*free*™ Kit (Ambion, Thermo Fisher Scientific, Waltham, MA, USA)
	use of intron-spanning primers if possible
	verification of amplicon-specificity via dissociation curve
CONCENTRATION	NanoDrop® ND-1000 spectrophotometer (Thermo Fisher Scientific, Waltham, Ma, USA)
	**Assay optimisation – validation**
ACCESSION NUMBER	see Supplementary Table S1 & S2
AMPLICON DETAILS	see Supplementary Table S1 & S2
PRIMERS SEQUENCE	see Supplementary Table S1 & S2
IN SILICO	TAIR primer-BLAST http://www.arabidopsis.org/Blast/index.jsp
EMPIRICAL	primer concentration of 300 nM unless stated otherwise (Table S1 & S2)
	annealing temperature of 60°C
PRIMING CONDITIONS	combinations of random hexamers and oligo-dT primers
PCR EFFICIENCY	dilution curves of pooled samples (slope, deviation)
LINEAR DYNAMIC RANGE	samples are within the range of the efficiency curve
	**RT and qPCR**
PROTOCOLS	Turbo DNA-*free*™ Kit (Ambion, Thermo Fisher Scientific, Waltham, MA, USA)
	PrimeScript™ RT Reagent Kit (Perfect Real Time, Takara Bio Inc., Kusatsu, Japan)
	Qauntinova™ SYBR® Green PCR kit (Qiagen, Hilde, Germany)
	as described in the Materials and Methods section
REAGENTS	as described in the Materials and Methods section
NTC	verification based on Cq-value and dissociation curve
	**Data analysis**
SPECIALIST SOFTWARE	7500 Fast System Sequence Detection Software, version 1.4.0 (Applied Biosystems, Thermo Fisher Scientific, Waltham, MA, USA)

STATISTICAL JUSTIFICATION	specified in Materials and Methods section and legends of Figures and Tables
NORMALISATION	a selection of at least three reference genes based on the GrayNorm algoritm (Remans *et al.* 2014)<br>see Materials and Methods section
